# Clinical Trials with Mesenchymal Stem Cell Therapies for Osteoarthritis: Challenges in the Regeneration of Articular Cartilage

**DOI:** 10.3390/ijms24129939

**Published:** 2023-06-09

**Authors:** Diego de Carvalho Carneiro, Lila Teixeira de Araújo, Girlaine Café Santos, Patrícia Kauanna Fonseca Damasceno, Jaqueline Leite Vieira, Ricardo Ribeiro dos Santos, Josiane Dantas Viana Barbosa, Milena Botelho Pereira Soares

**Affiliations:** 1 Gonçalo Moniz Institute, Oswaldo Cruz Foundation, Salvador 40296-710, Bahia, Brazil; diegoccarneiro@gmail.com (D.d.C.C.); lila.araujo@fieb.org.br (L.T.d.A.);; 2 SENAI Institute of Advanced Health Systems, University Center SENAI CIMATEC, Salvador 41650-010, Bahia, Braziljosianedantas@fieb.org.br (J.D.V.B.)

**Keywords:** clinical trials, osteoarthritis, mesenchymal stem cells, intra-articular injection, cartilage regeneration

## Abstract

Osteoarthritis (OA) is a whole-joint disease primarily characterized by the deterioration of hyaline cartilage. Current treatments include microfracture and chondrocyte implantation as early surgical strategies that can be combined with scaffolds to repair osteochondral lesions; however, intra-articular (IA) injections or implantations of mesenchymal stem cells (MSCs) are new approaches that have presented encouraging therapeutic results in animal models and humans. We critically reviewed clinical trials with MSC therapies for OA, focusing on their effectiveness, quality, and outcomes in the regeneration of articular cartilage. Several sources of autologous or allogeneic MSCs were used in the clinical trials. Minor adverse events were generally reported, indicating that IA applications of MSCs are potentially safe. The evaluation of articular cartilage regeneration in human clinical trials is challenging, particularly in the inflammatory environment of osteoarthritic joints. Our findings indicate that IA injections of MSCs are efficacious in the treatment of OA and the regeneration of cartilage, but that they may be insufficient for the full repair of articular cartilage defects. The possible interference of clinical and quality variables in the outcomes suggests that robust clinical trials are still necessary for generating reliable evidence with which to support these treatments. We suggest that the administration of just-sufficient doses of viable cells in appropriate regimens is critical to achieve effective and durable effects. In terms of future perspectives, genetic modification, complex products with extracellular vesicles derived from MSCs, cell encapsulation in hydrogels, and 3D bioprinted tissue engineering are promising approaches with which to improve MSC therapies for OA.

## 1. Introduction

Osteoarthritis (OA) is a disease that affects the integrity of diarthrodial joint tissues and is primarily characterized by the deterioration of hyaline cartilage [1,2]. OA is associated with aging, obesity, inflammation, and traumatic injuries, leading to progressive changes in the composition of the articular cartilage that result in structural and functional alterations. This whole-joint disease is generally caused by cellular senescence, inflammatory catabolism, and detrimental biomechanical modifications of articular tissues. OA is common in the elderly population and can progressively lead to disability if not properly treated [3].

Personalized management is a necessary strategy with which to treat this multifactorial disease. Evidence-based guidelines have been established by important medical organizations. They include several pharmacological and non-pharmacological therapies, which are commonly associated with self-management approaches [2]. The most common pharmacological therapies are the oral administration of non-steroidal anti-inflammatory drugs (NSAIDs) and selective inhibitors of cyclooxygenase 2 (COX2). Nutraceuticals containing glucosamine and chondroitin sulfate are recommended by some organizations. Exercise, dietary advice, and weight loss are non-pharmacological treatments that can improve physical function and reduce pain as well as the progression of cartilage degeneration. These treatments can be conditionally recommended, and they usually address the anatomical location of the disease in addition to the number of affected joints. Intra-articular injections of corticosteroids and hyaluronic acid in viscosupplementation are commonly recommended for knee OA [2,3,4]. 

Mesenchymal stem cells (MSCs) are non-hematopoietic, multipotent, and adult stem cells that can be obtained from various tissues, such as bone marrow, adipose, and umbilical cord tissues [5]. In addition to their ability to differentiate into lineages of the three germ layers, MSCs are perivascular cells that assume trophic as well as immunomodulatory functions when activated and establish regenerative microenvironments in injured tissues [6]. Cell–cell interactions in cellular aggregates of 3D structures play a critical role during the in vitro and in vivo chondrogenesis of MSCs. Several scaffolds have been studied to support these 3D structures in cartilage engineering, especially natural polymers that are components of the cartilage extracellular matrix, such as hyaluronic acid, collagen, and chondroitin sulfate. Hyaluronic acid has been reported to be the best-performing chondrogenic scaffold, via inducing the formation of hyaline-like cartilage tissues with coverage of chondral defects [7]. The application of MSCs in the regeneration of articular cartilage has garnered growing interest. It is a promising strategy, since it has demonstrated favorable results from bench to bedside; however, more translational efforts are necessary to successfully implement this approach with OA patients [8], which is especially important as the world population ages and rates of obesity as well as traumatic knee injuries increase [9].

The assessment of joint functionality for clinical and research purposes is essential for validating the outcomes of new treatment approaches. These outcome measurements include patient-reported outcome measures (PROMs), histological analyses, macroscopic repair, magnetic resonance imaging (MRI), clinical analyses, and functioning evaluations to measure progression to disability [10]. Although MRIs of hip joints have shown reliability and are recommended for use in the assessment of OA progression as well as recovery, they need the further technical and quantitative validation of reference measures [10]. Histological analyses are a benchmark for articular cartilage repair assessments, and several scoring systems have been proposed. The International Cartilage Repair Society (ICRS) recommends a cartilage repair grading system of core biopsies for studies concerning both animal models and human clinical trials, but a reference standard measure for the validation of the scores is still undetermined. Since these methods require a surgical procedure, histological and arthroscopic measures are inconvenient for human trials [11,12].

So far, pharmacological agents can only postpone tissue deterioration in OA. Total joint replacement is the last suitable intervention for end-stage degenerative OA, but early surgical strategies with which to repair osteochondral lesions are also available, such as microfracture (MF), autologous chondrocyte implantation (ACI), autologous matrix-induced chondrogenesis (AMIC), and matrix-assisted autologous chondrocyte implantation (MACI) [13]. The abovementioned cell-based cartilage defect repair strategies, as well as cell-free approaches, such as osteotomy, graft transplantation, and the implantation of scaffold biomaterials, are often inevitable therapies because of the limited self-regenerating ability of the articular cartilage tissue [14,15]; however, it is difficult to obtain durable, biomimetic, and weight-bearing hyaline-like cartilage repair for these surgical interventions. Therefore, there is an unattained need for new developments in the treatment of OA to prevent the progression of cartilage degeneration [13,14,15]. The objective of this article is to review clinical trials that used MSC therapies in the treatment of OA, aiming to evaluate their outcome measures, effectiveness, and quality in the regeneration of articular cartilage.

## 2. Mesenchymal Stem Cell Therapies

**Figure 1:**
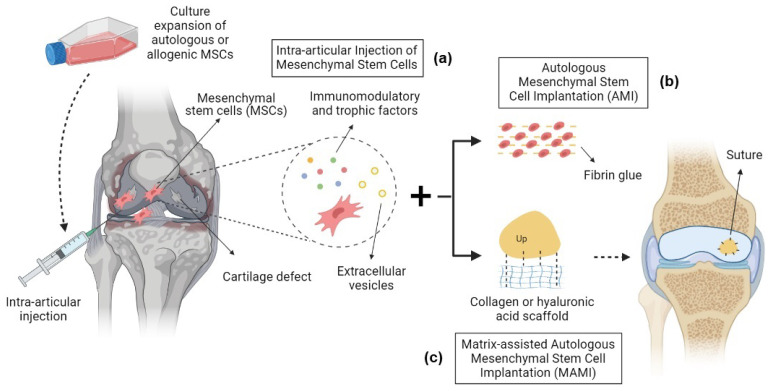
Mesenchymal stem cell therapy strategies in the treatment of OA. (**a**) Intra-articular injection of MSCs: autologous or allogeneic culture-expanded MSCs are harvested and injected into the articular cavity, where they produce immunomodulatory factors, trophic factors, and extracellular vesicles that induce cartilage defect regeneration and the alleviation of symptoms. (**b**) Autologous mesenchymal stem cell implantation (AMI): autologous culture-expanded MSCs are implanted back into cartilage defects with fibrin glue. (**c**) Matrix-assisted autologous mesenchymal stem cell implantation (MAMI): a bioscaffold is applied to the cartilage defect with culture-expanded MSCs.

Culture-expanded MSCs exhibit modifications in cell morphology, physiology, and gene expression profile, as well as increased risk of contamination by microorganisms and xenobiotics. Adverse events of MSC therapies may be associated with the consequences of culture expansion and unfavorable cell differentiation, especially for systemic intravascular administrations [16]; however, MSCs have been widely used in preclinical animal models to study their chondrogenic differentiation and signaling activities for the treatment of OA, especially in scaffold-free intra-articular (IA) injections. The benefits observed in these experimental studies motivated the progression of MSC therapy into human clinical trials [17]. IA injections of mesenchymal stem cells have presented encouraging outcomes for the treatment of OA in clinical trials, inducing the regeneration of articular cartilage defects and alleviating symptoms with safety as well as efficacy (Figure 1a) [18]. This approach has significant translational potential, but an optimum MSC formulation is still elusive, and larger in addition to more rigorous studies are needed before efficacious protocol therapies can be developed for usage in clinical routines [18].

### 2.1. Bone-Marrow-Derived Mesenchymal Stem Cells

A pilot study for MSC therapy in OA showed significant improvements in pain relief, as well as knee function and repair. IA injections of bone-marrow-derived MSCs (BM-MSCs) have been suggested as a promising treatment alternative that does not require hospitalization or surgery [19]. A phase I/II trial has shown evidence of cartilage restoration, pain mitigation, and the recovery of physical activities without serious adverse effects; however, this study lacked randomized control groups, longer follow-up times, and more relevant outcome measures [20].

A prospective, randomized controlled clinical trial with BM-MSC intra-articular injections, in participants undergoing high tibial osteotomy (HTO), demonstrated improvements in outcome scores compared to HTO alone [21]. About 61 million (M) culture-expanded autologous BM-MSCs injected into the joints of patients with knee OA demonstrated significant PROM improvement after 2 years, but just a slight increase in cartilage thickness after 1 year [22]. A five-year study with only three participants with moderate to severe OA showed limited outcome assessments, but suggested a short-term (6 months) improvement for injections of BM-MSCs into the knee joints. Although the results were higher than the baseline, deterioration was reported during the 5-year follow-up [23]. A larger study with a 4-year follow-up randomized 27 patients into either HA or HA with BM-MSC injections into the knee joint. This trial demonstrated clinical and functioning improvements in the group receiving BM-MSCs, but these long-term improvements were not sustained in the group receiving only HA [24].

A robust non-randomized study, including patients with late-stage knee OA treated with BM-MSCs at doses of 1, 10, and 50 M cells, confirmed the safety of MSC therapy by IA injection, even at high doses, while showing that it also decreased synovial inflammation and improved PROMs during a short-term follow-up [25]. Although cartilage catabolic biomarkers were significantly reduced, no changes in MRI T2 values were observed, indicating that the regenerative effects of MSC therapy are ineffective in late-stage OA, but that it may be efficacious for mild conditions [25]. IA injections of BM-MSCs were associated with platelet-rich plasma (PRP) in a controlled, double-blind clinical trial. This study suggested that it could improve the joint function and decrease symptoms of patients with knee OA when compared to the corticosteroid intervention at a 12-month follow-up; however, PRP only significantly enhanced KOOS pain subscores and did not provide additional benefits [26,27].

In randomized clinical trials, 30 subjects were allocated into two groups and received allogeneic BM-MSCs as well as HA (control) injections into their knee joints. This procedure resulted in minor adverse events, although pain, discomfort, inflammation, and swelling were common in the first week for both groups. Significant pain relief and a reduction in poor cartilage areas were observed [28]. In a preclinical and clinical trial, allogeneic cultured and pooled BM-MSCs (Stempeucel^®^) were injected into the knee joints of human participants allocated in dose groups with 25, 50, 75, or 150 million cells, followed by the administration of 10 mg/mL of HA. The best performance was observed in the 25 M group, with a 64.8% decrease in the WOMAC score compared to a reduction of 14.4% and 49.3% in the 50 M and placebo groups, respectively. Their results showed a trend for improvement, even though they were not statistically significant. MRI WORMSs did not show a difference from the baseline [29].

After three days of the subcutaneous administration of granulocyte colony-stimulating factor (G-CSF), BM-MSCs were harvested from the patients’ iliac crest, isolated, and filtered to obtain a stem cell concentrate with few blood cells [30]. This was followed by an immediate one-step IA injection of MSCs into the knee joint. The patients reported bone pain during G-CSF stimulation and moderate adverse effects for the BM-MSC injection. VAS scores significantly improved after 6 months for the treatment group, while no differences were observed in the group treated with paracetamol (control); however, there was no improvement in the WOMAC scores for either group [30].

Bone marrow aspirate concentrate (BMAC) is obtained via a procedure that harvests a concentrated fraction of MSCs and other progenitor cells from the iliac crest along with cytokines and growth factors [31]. BMAC intra-articular injections were initially demonstrated to be safe and viable as a cellular product in the treatment of OA; however, the outcomes of the BMAC- and saline-treated contralateral knees were similar [32]. Another randomized trial for OA also showed no significant differences between treatment and saline groups for pain relief and cartilage regeneration [33]. Sixty participants affected by the same OA grades in both knees were treated with the same MSC concentrations of BMAC injections in one knee joint, or in the subchondral bones of the contralateral knee. Interestingly, PROM and MRI scores were higher on the knee that received the subchondral bone injection [34].

Similarly, compared to total knee arthroplasty (TKA), BMAC subchondral injections in the contralateral knee of elderly patients led to the regression of bone marrow lesions over 2 years and were able to postpone or avoid TKA via pain mitigation after 10 years [35]. Therefore, in contrast to culture-expanded MSCs, BMAC is a complex product with a lower number of MSCs. Even though other cell types and growth factors may play a therapeutic role, BMAC therapy still require additional studies in order to understand the roles of other agents, to develop more efficacious formulations as well as dosage regimens, and to assess its viability over culture-expanded MSC therapy before regular application in the treatment of OA.

Chondrocytes, with their native pericellular matrix (chondrons), obtained from the debrided cartilage defects of mini-arthrotomy surgery procedures, were implanted with allogeneic BM-MSCs into cartilage defects in the knee of patients in a prospective clinical trial. A mixture of either 90% or 80% of MSCs presented trophic effects via the stimulation of those recycled chondrons to regenerate hyaline-like cartilage [36,37]. Using DNA short tandem repeat analyses of the regenerated cartilage biopsies, after 12 months the repaired tissue was shown to have only autologous chondrocytes and no allogeneic MSCs or chondrocytes differentiated from the MSCs. This procedure was safely performed in a one-stage surgery, instead of the common two-stage approach used in ACI treatments. This suggests that it may be more cost-effective and may prevent donor site morbidity in the repair of large defects [36,37]. This study demonstrated a significant improvement in clinical outcome scores in short- and mid-term follow-ups. It also showed an absence of serious adverse events, supporting its safety, potential efficacy, and longevity; however, due to the lack of randomization and a control group, it is not possible to evaluate if the improvements reported are certainly related to the intervention, or if they occurred by chance. This being the case, a randomized, blinded, and controlled study is strongly recommended in this situation [38].

### 2.2. Adipose-Derived Mesenchymal Stem Cells

Adipose-derived mesenchymal stem cells (AD-MSCs) have also been demonstrated to be safe for intra-articular therapy, even at a high concentration of 100 million cells. The patients reported a reduction in pain, and MRI, arthroscopy, and histological evaluations indicated a decrease in defect size as well as the regeneration of hyaline-like articular cartilage well-integrated onto the subchondral bone [39]. Later, another study showed no systemic or safety concerns in laboratory tests, vital signs, and electrocardiograms for dosages of up to 50 M cells. Pain, function, and mobility improved independently of the dose, while histological analyses indicated that most patients presented an absence of mild or moderate synovial inflammation and no signs of tumor proliferation [40]; however, these clinical trials were not randomized, lacked a control group, and had a short follow-up period. Therefore, this study did not identify long-term adverse events. More recently, a randomized, double-blinded, and placebo-controlled trial demonstrated that a single IA injection of AD-MSCs satisfactorily reduced symptoms related to OA after 6 months without relevant adverse events. The WOMAC total score was reduced by 55%, while cartilage defects remained unchanged in the MSC group and decreased in the saline control [41].

The efficacy of one or two AD-MSC dosing regimens of 100 M cells by IA injection was assessed in a randomized clinical trial, comparing it to conventional and conservative management approaches as controls. This study demonstrated that stem cell therapy surpassed the conventional methods by showing significant improvements in pain and function, as well as more consistent OA stabilization for the two-dose regimen with an interval of 6 months [42]. Stromal vascular fractions (SVFs) with AD-MSCs from the adipose tissues of 37 patients were injected into their knee joints in a study with double-blinded randomization into a placebo group and two groups treated with low and high SVF doses. SVFs were demonstrated to be safe and efficacious against OA symptoms and pain in a dose-dependent manner, but no changes in cartilage thickness were observed via MRI analyses [43].

A study showing preclinical and clinical results demonstrated that human AD-MSCs subcutaneously inoculated in BALB/c-nu nude mice led to abnormal manifestation, organ damage, and death; however, IA injections did not lead to serious adverse events and significantly reduced pain as well as improving knee function and cartilage volume [44]. The Re-Join^®^ product is composed of in vitro expanded autologous AD-MSCs. It presented a slight improvement in knee function, pain, and cartilage thickness compared to baseline and HA control in a phase II randomized, double-blinded study, but these outcomes were only apparent given the high variability of the data [45]. Another phase II study on Re-Join^®^ was conducted with randomized, single-blinded MF, MF with HA, and MF with HA and Re-Join^®^ treatment groups for 2 years. Using several assessments, including PROM, arthroscopic, MRI, and histological analyses, the treatment with Re-Join^®^ demonstrated a considerable reduction in articular cartilage defects and an improvement in joint function at 6- and 24-month follow-ups, respectively, without serious adverse events [46]; however, the study had a small number of participants in each group (7–10), a group treated only with Re-Join^®^ without other treatment approaches to provide direct evidence of efficacy was not included, and the cartilage regeneration outcome measures did not present sufficient statically significant evidence [46]. The intra-articular injection of allogeneic AD-MSCs (AlloJoin^®^) has been demonstrated to reduce pain and improve function in osteoarthritic knees in a phase I pilot study [47]. Above 70% of the participants presented at least 1 adverse event. Although they reported improvements in OA symptoms, a suggested slight improvement in cartilage volume was only observed for the indicated low-dose group (10 million cells) [47].

A large randomized clinical trial compared two treatment groups for knee OA: group 1 received a treatment of MF, followed by arthroscopic autologous mesenchymal stem cell implantation (AMI) of AD-MSCs (5 × 10^6^ cells) with fibrin glue (Figure 1b), and group 2 received MF alone. Group 1 showed improvements in pain and symptom scores, but no differences for the other subscales. Complete or hypertrophic defect filling was observed in 65 and 40% of cases in groups 1 and 2, respectively [48]. Patients with varus knee OA undergoing HTO treatment were simultaneously treated with AD-MSC implantation alone (5 × 10^6^ cells) or AD-MSCs with allogeneic cartilage implantation (MSC-AC). This large randomized short-term study showed significant improvement in both groups after 1 year, but further improvement in the MSC-AC group only [49].

The cartilage repair outcome of allogeneic AD-MSCs was assessed by multi-compositional MRI techniques in a randomized study with 18 subjects. While PROM scores demonstrated improvement in symptoms, T1rho mapping was indicated as the most sensitive approach. This study lacked a control group, and MRI analyses were not complemented with direct assessments of cartilage repair [50]. Allogeneic AD-MSCs (ELIXCYTE^®^) demonstrated effectiveness in a patient-blind, randomized, and active control study, but 43.9% of patients experienced at least one mild adverse effect [51]. All groups with IA injections of HA or 16, 32, and 64 M cells showed a reduction in WOMAC pain scores after 24 weeks of the treatment. Total WOMAC, stiffness, and functioning limitation scores significantly decreased at follow-up week four compared to HA, indicating that the cellular treatments had early efficacious outcomes [51].

### 2.3. Synovium- and Peripheral-Blood-Derived Mesenchymal Stem Cells

Synovium-derived mesenchymal stem cells (SD-MSCs) were applied in a matrix-assisted autologous mesenchymal stem cell implantation (MAMI) (Figure 1c), and this approach was compared to MACI in a short-term, prospective, and single-blinded randomized study. Without adverse events, MAMI demonstrated better functional and PROM outcomes, as well as good to excellent cartilage defect filling upon MRI evaluation. In addition, SD-MSCs were indicated to be more chondrogenic and less osteogenic than BM-MSCs, and they could be easily isolated and expanded in vitro [52]; however, histological and arthroscopic analyses were not performed in this study, so the characteristics of the regenerated cartilage tissue from MAMI were not compared to MACI, and no evidence of histological superiority could be certified [52]. A randomized and controlled trial used peripheral-blood-derived mesenchymal stem cells (PBMSCs) and HA after surgery via arthroscopic subchondral drilling in the knee. Once per week, post-surgery injections were performed during the first 5 weeks, after which patients then received weekly injections for 3 weeks at 6-, 12-, and 18-month follow-ups [53]. After two years, the treatment group receiving PBMSCs demonstrated considerable, significant improvement in clinical and radiological scores for massive chondral defects, compared to a control group that received physiotherapy and HA injections [53].

### 2.4. Umbilical-Cord- and Placenta-Derived Mesenchymal Stem Cells

A phase I/II clinical trial tested a composite of allogeneic human umbilical-cord-blood-derived mesenchymal stem cells (hUCB-MSCs) and HA hydrogel for intra-articular injections in seven participants. No adverse or undesired effects were observed, and patients reported a significant improvement in PROM scores at 6 months [54]. The ICRS arthroscopic and histological evaluations demonstrated the repair of a mature and well-integrated hyaline cartilage tissue after 3 months and 1 year of treatment, respectively. These improvements were maintained over 7 years, demonstrating effectiveness and durability for the regeneration of articular cartilage [54]. Improvements were also observed for hUCB-MSCs with HA treatments in a single-arm open-label clinical trial [55]. Placenta-derived mesenchymal stem cells (PLMSCs) were also studied in double-blinded and placebo-controlled studies. This study demonstrated clinical improvement at 2 months as well as an increase in cartilage thickness at 6 months [56].

## 3. Adverse Events of MSC Therapies in OA

Adverse events (AEs) must be properly described in clinical trials, and their evaluation, according to type, duration, intensity, and the number of patients (N), is very important for demonstrating reliable safety data to support a therapy. Twenty-one studies presented descriptions of AEs, eight reported that no AEs occurred during the clinical trial, and six did not show data for AEs. As can be seen in Table 1, the most common AEs were joint pain and swelling (effusion) beginning right after an IA injection of MSCs. The number of mild and moderate AEs was 89% and 11%, respectively. No MSC-therapy-related severe AEs were found. Although the duration of an AE is an important parameter to assess the safety of a treatment, 57% of the clinical trials did not show these data. Overall, the studies demonstrated that MSC therapies for OA were safe during their respective periods of follow-up.

## 4. Clinical Variables and Outcomes

**Figure 2:**
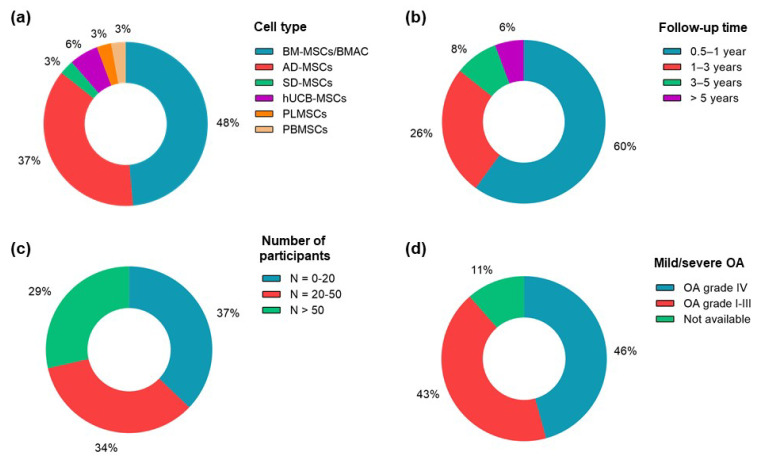
Main clinical variables of the clinical trials (total = 35). (**a**) Number of studies for each tissue source of MSCs. (**b**) Number of studies with short-, mid-, and long-term follow-ups. (**c**) Number of studies with small, average, and large numbers of participants. (**d**) Number of studies presenting participants diagnosed with mild (I–III) or severe (IV) OA grades.

The main variables and outcomes of the clinical trials are described in Table 2. Mild adverse events predominated in all of the clinical trials reviewed. Autologous (66%) or allogeneic (20%) BM-MSCs and AD-MSCs were the cell type choices in 49% and 37% of the clinical trials (Figure 2a), respectively, indicating that there is unexplored therapeutic potential of MSCs from other sources. Only 5 studies carried out 3- to 5- and greater than 5-year follow-ups (Figure 2b), and those within 1 year accounted for 60% of the clinical trials; however, the heterogeneity identified in these studies does not provide enough evidence for long-lasting regenerative and therapeutic effects.

Most trials did not discriminate the outcomes by the individual characteristics of the patients, such as age or obesity, although these variables are among the most important risk factors for OA [2]. In addition, only four clinical trials applied two-dose IA injections of MSCs instead of one-dose regimens, and 77% of the studies used autologous MSCs. A significant number of patients (N) participated in phase I and II studies (71%), but no phase III clinical trials have been described so far. Large clinical trials (with N > 50) were performed by 29% of the studies (Figure 2c), indicating that few studies presented more reliable statistical significance for the treatment of OA accounting for population variability. Figure 2d shows that most studies (46%) included participants with evidence of grade-IV OA, indicating that MSC therapies may have the potential to treat severe OA, but none of them compared the outcomes of different OA grade groups.

**Figure 3:**
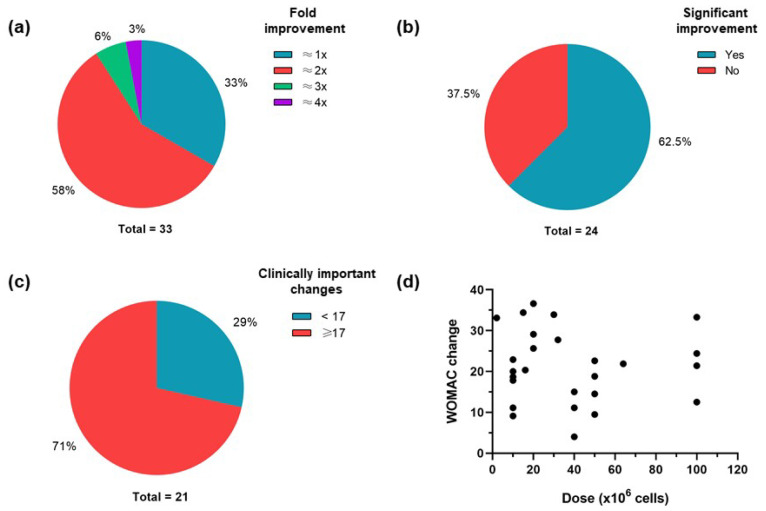
Assessment of the main outcomes described in the clinical trials. (**a**) Number of studies for each fold improvement of outcome scores. (**b**) Number of studies with or without significant MRI improvement. (**c**) Number of studies with minimum clinically important WOMAC score changes (≥17). (**d**) WOMAC score changes presented by IA-injected MSC doses from 15 studies.

As shown in Figure 3a, 67% of the studies presented PROM outcome scores with a fold improvement greater than 2×, while 62.5% of the clinical trials with MRI outcomes reported significant improvement (Figure 3b). Figure 3c shows that 71% of the studies presented WOMAC changes greater than 17 points; this analysis is based on the minimum clinically important difference for cohorts with baseline and follow-up WOMAC total scores of patients that received total knee arthroplasty treatment [57]. These findings suggest that IA injections of MSCs are efficacious in the treatment of OA as well as the regeneration of cartilage, but that they may be insufficient for the full repair of cartilage defects. In addition, no dose-dependent improvement was observed for WOMAC changes in 15 studies (Figure 3d), suggesting that other variables are possibly associated with its clinical efficacy.

## 5. Quality Assurance of Clinical Trials and MSC Manufacturing

Assuring quality in clinical trials, as well as the quality control and reproducibility of MSC production, are essential steps for the meaningful development and evaluation of a cell therapy. Clinical studies must follow a rigorous methodology and avoid bias, since reliable evidence in these studies is essential for supporting a decision or recommendation of a therapy. In this review, we evaluated 35 clinical trials and found that 85.7% and 80.0% of them described, in detail, the inclusion as well as exclusion criteria and the intra-articular injection or surgical procedures, respectively.

In addition to MSC therapies, most studies (71.4%) did not use other concomitant interventions, such as intra-articular injections of hyaluronic acid, microfracture, chondrocyte implantation, osteotomy, and matrix-assisted implantation; however, 8.6% of the clinical trials described the fact that patients used other medications during the study, while 51.4% did not mention the use of concomitant medication. Studies that perform other concomitant interventions or allow the use of anti-inflammatory as well as pain medications by patients during the clinical trial end up adding variables to the outcomes, which eventually produce bias and compromise confidence in the results.

The inclusion of a control group, randomization, blinding, and matching are also important criteria with which to assess the power in the generation of evidence and reliability of clinical trials. About 54.3% of the studies did not include a control group, i.e., they only compared the follow-up outcomes to the baseline measures. Randomization is essential to guarantee balance between the intervention and control groups, and was carried out in 62.9% of the studies. Blinding was found in 51.4% of the clinical trials, but matching was seen in only one study (2.9%), showing the association of outcomes for mechanisms of injury and combined procedures [48]. Randomization, blinding, and matching are important parameters with which to maintain homogeneity in the groups and reinforce the fact that the differences between them can be stronger associated with the intervention(s).

Cellular identity, purity, sterility, viability, and potency are essential features in quality control analyses for the manufacturing of MSCs and their therapeutic applications. These specifications are recommended in the guidelines and product-specific requirements of regulatory agencies for the qualification of cell therapy products, but there are no standards for quality control as of yet; however, the International Society for Cellular Therapy (ISCT) has established minimal criteria for the definition of MSCs that are pivotal in assuring quality, reproducibility, and reliability in their manufacturing as well as therapeutic applications [58].

Finally, we found that 89% of the clinical trials described the MSC manufacturing method, while 83% performed recommended quality control tests. These data indicate that most studies followed minimal criteria for the therapeutic use of MSCs. Altogether, these findings suggest the necessity for more clinical trials that clearly avoid bias from the use of concomitant medication by patients. It is also still necessary to include control groups, blinding, and matching in the study designs to generate reliable evidence for the application of MSCs in the treatment of OA.

## 6. Conclusions

Our review demonstrates that IA injections of MSCs are potentially safe and efficacious medicinal products, acting as signaling cells in the treatment of OA by creating regenerative microenvironments in the joints through the production of immunomodulatory and trophic factors [6]. The clinical trials reviewed in this article suggest that these factors are not enough to fully regenerate cartilage defects, but it is still elusive as to whether dose and dose regimens can be improved for more successful treatments [59]. Our findings also suggest the possible interference of clinical and quality variables in the regeneration of articular cartilage outcomes described in the reviewed studies, so more robust clinical trials are still necessary in order to generate reliable evidence and support a decision or recommendation for the application of MSCs in the treatment of OA.

While the number of IA-injected MSCs may not be correlated with outcome improvements, low and short-lasting cellular viability in joints may compromise the results of these treatments, so the administration of just-sufficient doses of viable cells in two or more regimens with appropriate intervals is critical in achieving effective and durable effects. In fact, superior clinical outcomes were demonstrated by repeated doses of MSCs in IA injections compared to a single-dose regimen [59]. The tissue source of MSCs can play a major role in the effectiveness of MSC therapies, but no ideal source, dose, preparation, or particular characteristics of MSCs have been drawn in the treatment of OA so far [60]. It is essential that future clinical trials enlighten these parameters by comparing monotherapies with different types of MSCs, following a rigorous scientific method.

The trophic activity of MSCs may also come from direct interactions and communication between MSCs and chondrocytes through gap junctions, such that intra-articular injections may not be supporting these necessary cell–cell interactions for the regeneration of articular cartilage [61]. Some clinical trials have demonstrated the fact that allogeneic MSCs are also potentially safe and effective, such that they may provide a better option for product escalation, standardization, and distribution since they can be readily available for clinical applications. MSCs have multiple effects and greater versatility as medicinal products compared to traditional treatments for OA. MSC therapies cannot be considered as definitive and effective solutions to this disease so far, as is also the case for the current available pharmacological and non-pharmacological therapies, but they present large potential for improvements as a single option in IA injections, and especially in association with other approaches.

PROM and MRI analyses were the standard reference measures of treatment efficacy, but the World Health Organization recommends the use of the International Classification of Functioning, Disability and Health (ICF) as an additional measure. The ICF includes the influences of environmental factors, shifting the attention to impacts instead of causes, and has been increasingly implemented in many settings [62]. We believe that the ICF can considerably enrich clinical studies of treatments for OA; therefore, future clinical trials should use improved experimental designs with two or more regimens and focus on assessing the mid- and long-term effects of IA MSC therapy, age-associated efficiency, and functioning evaluations.

The heterogeneous nature of OA and the variability of study designs and quality of clinical trials may have contributed to the high variability in cartilage repair outcome measures seen in the clinical trials. Most studies showed an improvement in symptoms and functions via PROM scores, but non-invasive assessments do not show complete evidence of cartilage regeneration. MRI analyses and their outcome scores may be less realistic because they cannot completely assess the cartilage structure and lack a better validation of measures that associate the MRI evidence with clinical reality [10]. Macroscopic assessment scores via second-look arthroscopy and histological analyses are invasive, but provide better evidence for cartilage repair; however, this type of assessment is scarce in clinical trials, leading to incomplete analyses of cartilage regeneration in humans [11,12]. The demonstration of significant therapeutic improvement in cartilage structure by radiographic measures in clinical trials is still challenging, and the relationship between the regeneration of cartilage and relief of symptoms in OA treatments with MSCs remains unclear.

Several meta-analyses of clinical trials for stem cell therapies are available. These studies were suggested to present methodological flaws and should be interpreted with caution [63]. A recent descriptive systematic review has also found improvements in clinical and radiological outcomes of MSC therapies in randomized controlled trials, but their quality of evidence was reported as being low to very low [64]. These reviews reinforce the existence of a high heterogeneity of methods and data in the clinical trials with MSC treatments for OA, indicating that a consensus over this issue is still challenging and will still take time to reach; this being the case, it is urgent that future trials follow common guidelines. Since MSCs alone may not be sufficient to completely repair articular cartilage defects, it is also pressing that future clinical trials address more robust approaches, such as the use of genetically modified MSCs, intra-articular injections of MSCs encapsulated in hydrogels, and cartilage tissue engineering.

## 7. Future Perspectives

New strategies that reduce or reverse cellular senescence are promising since the world population is aging and cell senescence is an important contributor to poor cartilage repair. Autologous MSCs from elders may be a source of senescent chondrocytes, such that they would not be appropriate for cartilage bioengineering since they are likely to be less resistant to the degenerative environment of OA joints [65]. Induced pluripotent stem cells (iPSCs) can be better cell source options to produce more anabolic active articular cartilage tissues [66]. iPSCs or mesenchymal stem cells derived from iPSCs have not been used in clinical investigations for intra-articular injection treatments given their tumorigenic potential, but future developments in iPSC technology can overcome this issue [67].

IA injections of MSCs can be more complete sources of trophic factors instead of single factors isolated from them, but complex medicinal products composed of extracellular vesicles from culture-expanded MSCs are also promising non-cellular approaches for intra-articular injection treatments of OA [68]. The use of MSC-derived exosomes may be more advantageous since MSCs present risks of adverse events associated with their cell differentiation potential, but minimally manipulated and more differentiated cells can be an alternative with which to solve this problem [16].

The metallic or ceramic prosthesis used in total joint replacement surgeries for end-stage OA can lead to problems such as infection, instability, loosening, and stiffness [69], such that tissue engineering technologies, such as 3D bioprinting, are an auspicious approach with which to engineer bioprostheses of cartilage and bone tissues to replace those acellular materials [70]. The possibility of the control and development of scaffold structures in 3D bioprinting makes this technology a promising strategy with which to engineer more suitable articular cartilage tissues to repair defects or replace parts of the joints. Scaffolds based on molecules that naturally compose hyaline cartilage, such as gelatin networks linked to hyaluronan and chondroitin sulfate, have shown the ability to produce stronger stiff matrices and induce MSC chondrogenic differentiation [71].

Treatments that regenerate cartilage and decrease inflammation as well as pain without surgical procedures, as well as treatments with minimal, less invasive, or one-stage surgical procedures, are promising approaches for mild and moderate conditions. They avoid or reduce hospitalization, costs, and other issues associated with surgery, which positively influence the quality of life of these patients [37]. Cell harvesting and IA injections of MSCs are minimally invasive procedures, and they can be used in the treatment of OA to target the disease progression without surgery-associated risks of complications. The therapeutic properties of MSCs, progenitor cells, pluripotent stem cells, and genetically modified stem cells as well as chondrocytes have been reviewed as promising minimally invasive therapies [72]. The regeneration of cartilage is still challenging in OA, particularly in the inflammatory environment of osteoarthritic joints, such that new therapies that address joint inflammation are necessary. IA injections of MSCs may significantly advance OA treatments if they can fully regenerate articular cartilage defects with a hyaline-like tissue while reducing inflammation and pain [4,8].

Genetically engineered or reprogrammed MSCs may be a promising approach with which to enhance cell therapies to restore joint homeostasis [73]. Injections of MSCs encapsulated in biomaterials, such as hydrogels, may improve treatments via increasing their viability as well as the inter-cellular interactions between MSCs and chondrocytes. Advances in current clinical therapies have not yet solved some of the problems associated with these treatments, such as low clinical efficacy, the formation of unfit weight-bearing cartilage tissues, short longevity, the insufficient filling of large defects, and weak integrity to surrounding tissues. Therefore, MSC therapies are promising new options that can be associated with other clinical treatments to improve cartilage regeneration and joint healing [36,37].

## Figures and Tables

**Table 1:** MSC-therapy-related adverse events described in the clinical trials.

Reference	Follow-Up (Years)	AE	Number of Patients	Percentage of N	Duration of AE	Intensity of AE
[19]	1	Joint pain	6	50%	1–6 days	Mild
		Joint inflammation	3	25%	NA	Mild
		Back pain	3	25%	NA	Mild
		Tendonitis	1	8%	NA	Mild
[20]	1	Joint swelling	2	13%	NA	Mild
		Joint lock	1	6%	NA	Mild
		Back pain	3	20%	2–3 days	Mild
		Arthralgia	8	53%	2–3 days	Mild
[22]	1–2	Joint pain	2	15%	1 day	Mild
		Joint swelling	1	8%	2 days	Mild
[25]	1	Joint pain	4	33%	2–4 weeks	Mild
		Joint swelling	2	17%	4 weeks	Mild
[30]	0.5	Joint pain and swelling	1	2%	2 days	Mild
[32]	0.5	Joint swelling	3	12%	6 months	Moderate
[33]	1	Joint swelling	2	8%	12 months	Moderate
[40]	0.5	Joint swelling	5	28%	NA	Mild
[41]	0.5	Arthralgia	6	50%	6 months	Moderate
		Joint swelling	2	17%	6 months	Moderate
[42]	1	Joint pain and swelling	2	7%	4 weeks	Mild
		Discomfort and bruising	18	60%	NA	Mild
[43]	0.5–1	Joint swelling	1	3%	NA	Mild
[44]	2	Joint pain	4	11%	NA	Mild
		Joint swelling	15	44%	NA	Mild
		Joint edema and cramps	1	3%	NA	Mild
[45]	1	Joint pain and swelling	19	36%	7 days	Mild
[46]	2	Joint pain and swelling	5	17%	NA	Mild
		Skin erythema	2	7%	NA	Mild
[28]	1	Joint pain and swelling	8	53%	7 days	Mild
[29]	1	Joint pain and swelling	4	7%	NA	Mild
		Arthralgia	2	3%	NA	Mild
[47]	1	Joint pain	18	82%	3 weeks	Moderate
		Joint swelling	3	14%	3 weeks	Mild
		Joint edema	10	45%	3 weeks	Mild
[51]	1	Joint pain	9	16%	NA	Mild
		Joint swelling	6	10%	NA	Mild
		Arthralgia	8	14%	NA	Mild
		Joint stiffness	2	3%	NA	Mild
[54]	7	Arthralgia	2	30%	NA	Mild
		Back pain	1	14%	NA	Mild
		Increased infection susceptibility	1	14%	NA	Mild
[56]	0.5	Joint pain and swelling	4	20%	3 days	Mild
[37]	1.5	Joint swelling	8	23%	NA	Mild
		Arthralgia	13	37%	NA	Mild
		Crepitations	5	14%	NA	Mild

NA: not available.

**Table 2:** Main variables and outcomes of clinical studies with mesenchymal stem cell therapies.

Cell Type(s)	Treatment Approach	Dose (×10^6^ Cells)	Regimen (Interval)	Phase	Follow-Up (Years)	N	Age (years)	Defect Size (cm^2^)	OA Grade	Main Outcome Results		Reference
										PROM/Function	MRI/Arthroscopy	
Autologous BM-MSCs	Intra-articular injection	40	One dose	Pilot	1	12	44–54	NA	II–III	WOMAC ^a,b^: 19.4–8.3	MRI (PCI) ^a^: 19.5–15.4	[19]
Autologous BM-MSCs	Intra-articular injection	40	One dose	I/II	1	15	33–64	NA	II–III	WOMAC ^a,b^: 25–10	MRI T2 scores ^a^: 59.64–51.14	[20]
Autologous BM-MSCs	Intra-articular injection	13	One dose	NA	2	56	24–54	1.5–9.3	IV	IKDC ^a^: 33.9–85 (HTO + MSCs) with additional improvement of 7.65 compared to HTO	MRI (MOCART) ^c^: 43.21 (HTO) and 62.32 (HTO + MSCs) with *p* < 0.001	[21]
Autologous BM-MSCs	Intra-articular injection	30	Two doses (1 month)	I/II	1–2	13	34–63	NA	II–III	KOOS (symptoms) ^a^: 67.3–88.7	MRI ^a^: cartilage thickness: from 2.15–2.16 to 2.38–2.5 (mm)	[22]
Autologous BM-MSCs	Intra-articular injection	8–9	One dose	NA	5	3	54–65	NA	II–III	VAS ^a^: from 80, 85, and 90 to 45, 8, and 45, respectively	NA	[23]
Autologous BM-MSCs	Intra-articular injection (+ HA)	10 or 100	One dose	I/II	4	27	54–69	NA	II–IV	WOMAC ^a,b^: 27–27 (control), 37–17 (10 × 10^6^), and 29–16.5 (100 × 10^6^)	NA	[24]
Autologous BM-MSCs	Intra-articular injection	1, 10, or 50	One dose	I/IIa	1	12	40–65	NA	III–IV	NA	MRI: no changes for T2 scores and WORMSs	[25]
Autologous BM-MSCs	Intra-articular injection (+ PRP)	40	One dose	I	1	47	42–71	NA	I–IV	KOOS ^a,b^: 36.9–54.4 (corticosteroid), 30.3–54.2 (BM-MSCs), and 37.3–59.9 (BM-MSCs + PRP)	NA	[26]
Autologous BM-MSCs	Intra-articular injection	20	One dose	I/II	0.5	61	43–70	NA	II–III	WOMAC ^a,b^: 62.61–91.73 (BM-MSCs), and 69.93–72.96 (control)	NA	[30]
Autologous BMAC	Intra-articular injection	0.034	One dose	NA	0.5	25	42–68	NA	II–IV	VAS ^a^: 3.1–1.5 (BMAC) and 2.9–0.8 (saline) with *p* = 0.44	NA	[32]
Autologous BMAC	Intra-articular injection	0.034	One dose	NA	0.5–1	25	42–68	NA	I–III	VAS ^c^: 1.2 (BMAC) and 0.7 (placebo) with *p* = 0.98	MRI (T2 scores) ^c^: 2.4 (BMAC) and 2.5 (placebo) with *p* = 0.27	[33]
Autologous BMAC	Subchondral bone or intra-articular injections	0.114	One dose	NA	2	60	48–72	NA	I–IV	VAS ^a^: 4–1 (subchondral) and 3.5–3.5 (intra-articular)	Defect sizes (cm^2^) ^a^: from 0.4–5.2 to 1.4–2.9 (subchondral) and no regression (intra-articular)	[34]
Autologous BMAC	Subchondral bone injections	0.156	One dose	NA	2–10	140	65–90	NA	II–IV	VAS ^a^: 3.5–1.5 (BMAC), and 3.4–2.5 (TKA)	Cartilage volume increase of 2.3% at 2-year follow-up	[35]
Autologous AD-MSCs	Intra-articular injection	10, 50, or 100	One dose	I/II	0.5	18	54–72	2–6	III–IV	WOMAC ^a,b^: 54.2–32.8 (100 × 10^6^) and no improvement (10 and 50 × 10^6^)	Decrease of 40–51% (MRI) and 64% (arthroscopy) in hyaline cartilage defect size	[39]
Autologous AD-MSCs	Intra-articular injection	2, 10, or 50	One dose	I	0.5	18	57–74	NA	III–IV	WOMAC ^a,b^: 60.7–27.6 (2 × 10^6^), 47.2–24.3 (10 × 10^6^), and 38.8–16.2 (50 × 10^6^)	Possible cartilage improvement in three of six patients	[40]
Autologous AD-MSCs	Intra-articular injection	100	One dose	IIb	0.5	12	55–69	0.4–7	II–IV	WOMAC ^a,b^: 60.0–26.7	Defect sizes(cm^2^) ^a^: 3.12–3.15 (MSC) and 3.20–3.56 (control)	[41]
Autologous AD-MSCs	Intra-articular injection	100	One or two doses (6 months)	NA	1	30	44–65	NA	II–III	WOMAC ^a,b^: 59.0–60.0 (control), 59.6–84.0 (one dose), and 54.4–87.3 (two doses)	MRI (MOAKS): progression of cartilage loss in 67% (control), 30% (one dose), and 11% (two doses) of the participants	[42]
Autologous AD-MSCs (SVF)	Intra-articular injection	15 or 30	One dose	NA	0.5–1	37	41–74	NA	II–III	WOMAC ^a,b^: 47.1–13.2 (30 × 10^6^), 56.2–21.8 (15 × 10^6^), and 49.3–41.9 (placebo)	No significant difference in cartilage thickness between MSC and control groups	[43]
Autologous AD-MSCs	Intra-articular injection	10, 20, or 50	Two doses (12 months)	I/IIa	2	18	40–70	NA	II–III	WOMAC ^a,b^: 25.8–8.0 (10 × 10^6^), 49.0–12.4 (20 × 10^6^), and 31.2–12.4 (50 × 10^6^)	MRI ^a^: 23–125 mm^3^ (cartilage volume)	[44]
Autologous AD-MSCs (Re-Join^®^)	Intra-articular injection	50	One dose	IIb	1	52	45–64	NA	I–III	WOMAC ^a,b^: 30.83–21.35 (Re-Join^®^) and 34.17–27.25 (HA) with *p* < 0.0005	MRI: apparent overall increase in cartilage thickness	[45]
Autologous AD-MSCs (Re-Join^®^)	Intra-articular injection	50	One dose	IIa	2	30	52–70	1–8	III	WOMAC ^a,b^: 40.2–37.3 (MF), 40.9–31.0 (MF + HA), and 45.8–29.0 (MF + HA + Re-Join^®^)	ICRS-II ^a^: 28.1–27.4 (MF), 27.7–43.2 (MF + HA), and 32.0–55.9 (MF + HA + Re-Join^®^)	[46]
Autologous AD-MSCs	AMI	Not applicable	Not applicable	NA	2	80	32–46	3–7	III–IV	KOOS (symptoms) ^c^: 32.3 (group 1) and 27.8 (group 2) with *p* = 0.005	MRI (MOCART) ^c^: 62.4 (group 1) and 51.8 (group 2) with *p* = 0.033	[48]
Autologous AD-MSCs	AMI	Not applicable	Not applicable	NA	1–2	70	42–68	2.1–9.5	NA	KOOS (symptoms) ^c^: 67.3 (MSC) and 73.6 (MSC-AC) with *p* < 0.001	Higher Kanamiya grades in the MSC-AC group	[49]
Allogeneic BM-MSCs	Intra-articular injection	40	One dose	I/II	1	30	36–73	NA	II–IV	WOMAC ^a,b^: 45–41	MRI (PCI) ^a^: 14–9.5 (MSCs) and 15.5–12.5 (HA) with *p* < 0.05 at 1 year	[28]
Allogeneic BM-MSCs (Stempeucel^®^)	Intra-articular injection	25, 50, 75, or 150	One dose	II	1	60	47–67	NA	II–III	WOMAC ^a,b^: 1315.8–717.8 (25 × 10^6^), 1498.4–359.9 (50 × 10^6^), and 1239.6–233.8 (control)	MRI (WORMS) ^a^: 67.0–66.1 (25 M), 78.8–78.0 (50 M), and 76.5–74.9 (control)	[29]
Allogeneic AD-MSCs (AlloJoin^®^)	Intra-articular injection	10, 20, or 50	Two doses (3 months)	I	1	22	49–65	NA	II–III	WOMAC ^a,b^: 48.00–24.29 (10 × 10^6^), 42.13–25.63 (20 × 10^6^), and 40.14–29.43 (50 × 10^6^)	MRI ^a^: 10.34–54.58 mm^3^ of total cartilage volume increase (low dose)	[47]
Allogeneic AD-MSCs	Intra-articular injection	10, 20, or 50	One dose	I/IIa	1	18	40–70	NA	II–III	WOMAC ^a,b^: 38.83–24.33 (50 × 10^6^), 48.83–23.17 (20 × 10^6^), and 46.17–27.50 (10 × 10^6^)	MRI (T1rho) ^a^: 41.55–38.82 (high dose), 39.30–37.48 (mid-dose), and 38.91–37.94 (low dose)	[50]
Allogeneic AD-MSCs	Intra-articular injection	16, 32, or 64	One dose	I/II	1	57	51–79	NA	II–III	WOMAC ^a,b^: 41.50–25.75 (HA), 42.88–22.53 (16 × 10^6^), 46.41–18.65 (32 × 10^6^), and 35.27–13.40 (64 × 10^6^)	NA	[51]
Autologous SD-MSCs	MAMI	Not applicable	Not applicable	NA	2	14	18–46	2.1–4.3	NA	KOOS (symptoms) ^a,c^: 66.33–89.80 (MAMI) and 67.46–83.67 (MACI) with *p* = 0.015	MRI (graft infill, score 1–4)^a^: 2.93–3.86 (MAMI) and 2.64–3.29 (MACI) with *p* = 0.005 from 3 to 6 months	[52]
Allogeneic hUCB-MSCs	Intra-articular injection (+ HA)	12–20	One dose	I/II	0.5–7	7	29–77	4.6–8.1	III–IV	IKDC ^a^: 39.1–63.2 (6 months)	MRI ΔR1 index of 1.44 (3 years)	[54]
Allogeneic hUCB-MSCs	Intra-articular injection (+ HA)	10	One dose	NA	1	29	48–68	NA	I–IV	WOMAC ^a^: 22.55–13.46 (mild OA) and 27.57–16.42 (severe OA)	MRI (medial T2 map) ^a^: 58.72–62.58 (mild OA) and 201.57–68.97 (severe OA)	[55]
Allogeneic PLMSCs	Intra-articular injection	50–60	One dose	Pilot	0.5	20	NA	NA	II–IV	KOOS (symptoms) ^c^: 41.10 (PLMSCs) and 38.80 (control)	MRI ^a^: Increase in chondral thickness: 2.7 to 3.5 mm	[56]
Autologous PBMSCs	Intra-articular injection (+ HA)	NA	NA	IIb	2	120	23–55	≥3	III–IV	IKDC ^a^: 42.7–48.1 (control) and 43.1–65.6 (intervention)	MRI (MOCART) ^a^: 10.9–15.6 (control) and 13.1–54.0 (intervention)	[53]
Allogeneic BM-MSCs	ACI + BM-MSCs	Not applicable	Not applicable	I/II	1.5	35	22–38	2–5	NA	KOOS ^a,b^: 57.9–85.4	MRI (T1rho) ^c^: 43.1 (healthy control) and 47.9 (repaired cartilage)	[37]
Allogeneic BM-MSCs	ACI + BM-MSCs	Not applicable	Not applicable	I/II	5	35	22–38	2–5	NA	KOOS ^a,b^: 57.9–78.9	NA	[38]

NA: not available; a: means from the baseline to the follow-up; b: overall mean of subscales; c: only means of experimental and control groups; WOMAC: Western Ontario and McMaster Universities Osteoarthritis Index; KOOS: Knee Injury and Osteoarthritis Outcome Score; IKDC: International Knee Documentation Committee Scores; PCI: poor cartilage index; HTO: high tibial osteotomy; VAS: visual analog score; WORMSs: whole-organ MRI scores; HA: hyaluronic acid; BMAC: bone marrow aspirate concentrate; AMI: autologous mesenchymal stem cell implantation; MAMI: matrix-assisted autologous mesenchymal stem cell implantation; SVF: autologous stromal vascular fraction; MOAKS: MRI osteoarthritis knee scores; and PRP: platelet-rich plasma.

## Data Availability

No new data were created or analyzed in this study. Data sharing is not applicable to this article.
